# SLC52A3 expression is activated by NF-κB p65/Rel-B and serves as a prognostic biomarker in esophageal cancer

**DOI:** 10.1007/s00018-018-2757-4

**Published:** 2018-02-10

**Authors:** Lin Long, Xiao-Xiao Pang, Fei Lei, Jia-Sheng Zhang, Wei Wang, Lian-Di Liao, Xiu-E Xu, Jian-Zhong He, Jian-Yi Wu, Zhi-Yong Wu, Li-Dong Wang, De-Chen Lin, En-Min Li, Li-Yan Xu

**Affiliations:** 1 Department of Biochemistry and Molecular Biology, Shantou University Medical College, Shantou, China; 2 The Key Laboratory of Molecular Biology for High Cancer Incidence Coastal Chaoshan Area, Shantou University Medical College, Shantou, China; 3 Institute of Oncologic Pathology, Shantou University Medical College, Shantou, 515041 Guangdong China; 4 Department of Oncology Surgery, Shantou Central Hospital, Affiliated Shantou Hospital of Sun Yat-Sen University, Shantou, China; 5 Henan Key Laboratory for Esophageal Cancer Research, Department of Basic Oncology and Pathology at College of Medicine, The First and The Second Affiliated Hospital, Zhengzhou University, Zhengzhou, Henan China; 6 Guangdong Province Key Laboratory of Malignant Tumor Epigenetics and Gene Regulation, Research Center of Medicine, Sun Yat-Sen Memorial Hospital, Sun Yat-Sen University, Guangzhou, China

**Keywords:** SLC52A3, Riboflavin, TNFα, NF-κB, Rel-B, Esophageal cancer

## Abstract

The human riboflavin transporter-3 (encoded by *SLC52A3*) plays a prominent role in riboflavin absorption. Interestingly, abnormal expression patterns of SLC52A3 in multiple types of human cancers have been recently noted. However, the molecular mechanisms underlying its dysregulation remain unclear. In this study, we find that SLC52A3 has two transcript variants that differ in the transcriptional start site, and encode different proteins: SLC52A3a and SLC52A3b. Importantly, aberrant expressions of SLC52A3 are associated with stepwise development of esophageal squamous cell carcinoma (ESCC) as well as the survival rates of ESCC patients. Functionally, SLC52A3a, but not SLC52A3b, strongly promotes the proliferation and colony formation of ESCC cells. Furthermore, *SLC52A3* 5′-flanking regions contain NF-κB p65/Rel-B-binding sites, which are crucial for mediating *SLC52A3* transcriptional activity in ESCC cells. Chromatin immunoprecipitation and electrophoretic mobility shift assay reveal that p65/Rel-B bind to 5′-flanking regions of *SLC52A3*. Accordingly, NF-κB signaling upregulates SLC52A3 transcription upon TNFα stimulation. Taken together, these results elucidate the mechanisms underlying SLC52A3 overexpression in ESCC. More importantly, our findings identify SLC52A3 as both a predictive and prognostic biomarker for this deadly cancer.

**Electronic supplementary material:**

The online version of this article (10.1007/s00018-018-2757-4) contains supplementary material, which is available to authorized users.

## Introduction

Esophageal squamous cell carcinoma (ESCC) is the sixth most lethal malignancy in China, with an incidence of 21.62/10^5^ [1]. Nutritional imbalance has been suggested as one of the risk factors for ESCC [2–5]. Notably, a few reports indicated that lack of dietary riboflavin was associated with high risk for ESCC [6, 7]. However, the biological significance of riboflavin in the context of ESCC remains unknown. Many studies suggested that riboflavin played a role in immune system. Particularly, riboflavin deficiency affects cytokine production, and riboflavin-deprived cells released less IL-10 (anti-inflammatory) and more TNFα (pro-inflammatory) [8–11].

Human riboflavin transporter-3 (encoded by *SLC52A3*) is a trans-membrane protein that has been shown to play an important role in the absorption of riboflavin and regulation of riboflavin tissue distribution [12, 13]. Humans and other mammals (which lack the ability to synthesize riboflavin endogenously) obtain riboflavin from exogenous sources including dietary and normal microflora of large intestine [14]. SLC52A3 was reported as the most efficient transporter of riboflavin [15]. Notably, different groups found that riboflavin deficiency increased the expression of SLC52A3 [13, 16, 17], implying a negative feedback regulatory mechanism maintaining riboflavin homeostasis.

SLC52A3 has been implicated in the biology of several tumor types, including those from stomach and cervix [17–19]. It was also reported that SLC52A3 is upregulated in ESCC and glioma, compared with nonmalignant adjacent tissue. Moreover, SLC52A3 enhanced the proliferation of ESCC and glioma cells [20, 21]. However, little is known concerning how the expression of SLC52A3 is regulated at the transcriptional level in ESCC cells. More importantly, the clinical significance and biological relevance of SLC52A3 expression remain to be elucidated.

## Materials and methods

### Cell cultures and treatment

Cell lines used in this study were previously described [22, 23]. ESCC cell lines (KYSE150, KYSE180, KYSE510, and TE3) were maintained in 1640 medium (Thermo Fisher Scientific) containing 10% fetal bovine serum (GIBCO). SHEEC and SHEE cells were cultured in Dulbecco’s modification of Eagle’s medium Dulbecco (DMEM)/F12 medium (Thermo Fisher Scientific) with 10% newborn bovine serum (ExcellBiology). Immortalized esophageal epithelial cell lines (NE2 and NE3) were cultured in a 1:1 mixture of defined keratinocyte serum-free medium (Thermo Fisher Scientific) and EpiLife (Thermo Fisher Scientific). HEK293T cells were cultured in Dulbecco’s modified Eagle’s medium (Thermo Fisher Scientific) supplemented with 10% fetal bovine serum. All cells were incubated at 37 °C in a humidified atmosphere of 5% CO_2_ in air and maintained in media supplemented with penicillin-G (100 units/mL) and streptomycin (100 μg/mL).

In functional assays, KYSE150 and KYSE510 cells were seeded into 60 mm cell culture dishes at the density of 5 × 10^5^ cells per dish and treated with various concentrations TNFα (0, 20, 100 or 200 ng/mL, an NF-κB inducer, Promega) for 0, 3, or 6 h, respectively. In drug treatment experiments, cells were pretreated with QNZ (500 nM, a NF-κB inhibitor, Selleck) or JSH-23 (300 nM, an NF-κB inhibitor, Selleck) for 24 h, followed by TNFα (20 ng/mL, ab NF-κB inhibitor, Promega) treatment for 6 h.

### Sample collection and tissue microarray construction

For the retrospective survival analysis study, immunohistochemical staining was performed on ESCC tissue microarrays (TMA) constructed from paraffin-embedded specimens surgically resected at Shantou Central Hospital from 1987 to 1997 (246 cases) and 2007 to 2014 (290 cases). Tissue microarrays were constructed based on standard techniques as previously described [24]. Information on gender, age, stage of disease, and histopathologic characteristics was obtained from the medical records. Patients’ data were summarized in Supplementary Table S1 and Table S2. Data analysis indicated that there were no survival advantages associated with the use of radiotherapy or chemotherapy compared with the surgery-alone group, so we assessed the patients’ survival together. Kaplan–Meier survival evaluation revealed that the prognosis of patients was significantly associated with the histological grade, regional lymph node metastasis, and pTNM stage (*P* < 0.05). The patients who suffered from severe post-operative complications and those who died of other tumors or other causes were excluded. The study was approved by the governmental ethics committee.

To find out the expression pattern of SLC52A3 during the progression from normal esophagus to ESCC, 39 paraffin-embedded specimens resected at Shantou Central Hospital were selected for immunohistochemical analysis in the study. The normal esophagus and some lesions of hyperplasia, high-grade dysplasia, including all noninvasive neoplastic epithelia, previously called carcinoma in situ, and ESCC were evaluated in each sample. The normal epithelia were seen in 20 samples, while hyperplasia was present in 13 cases, lesions of high-grade dysplasia in 21 sections, and ESCC tissues in 37 of the 39 samples.

### SLC52A3a and SLC52A3b polyclonal antibody production

Rabbit anti-SLC52A3a and anti-SLC52A3b polyclonal antibody were produced by Zhoushan Bio-Technique Co., Ltd., Shandong, China. They used human SLC52A3a-unique sequence LRLFSSADFCNLHCPA (16 C-terminal amino acids) and SLC52A3b-unique sequence SIRPVGLLPLRTPHP (15 C-terminal amino acids) designed immunizing peptide and confirmed their stringent specificity using recombinant human SLC52A3 polypeptide by western blot.

### Immunohistochemical staining

Immunohistochemistry (IHC) was performed as described in our previous studies [25]. Briefly, sections 4 μm thick were dewaxed in xylene, rehydrated in alcohol, and incubated in 3% hydrogen peroxide for 10 min to block endogenous peroxidase activity. Sections were incubated with 10% normal goat serum in PBS for 15 min at room temperature to block nonspecific binding. Then sections were incubated overnight at 4 °C with primary antibodies for SLC52A3 antibody (1:50, Abgent), SLC52A3a antibody (1:50, Zhoushan Bio-Technique), or SLC52A3b antibody (1:50, Zhoushan Bio-Technique). After rinsing with PBS, slides were incubated for 10 min at 37 °C with HRP Polymer Conjugate (ZYMED, USA). Subsequently, slides were stained with 0.003% 3,3-diaminobenzide tetrahydrochloride and 0.005% hydrogen peroxide in 0.05 M Tris–HCl (pH 7.2), and counterstained with hematoxylin, dehydrated, and mounted. Positive reactions were defined as those showing brown signals in the esophageal squamous cell cytoplasm, nucleus or membrane. Each separate tissue core was scored on the basis of the intensity and area of the positive staining. The intensity of positive staining was scored: 0, negative; 1, weak staining; 2, moderate staining; 3, strong staining. The percentage of positive cells was scored on a 0–4 scale: 0, 0–5%; 1, 6–25%; 2, 26–50%; 3, 51–75%; 4, > 75%. The total score was obtained by multiplying the two scores above producing a total score range of 0–12. For statistical analysis, we treated all the samples with a total score of 0–4 as low (−) and of 5–12 as high (+).

### Mapping of the SLC52A3 gene transcriptional start sites (TSSs)

Total RNA from cell lines were extracted using TRIzol reagent (Invitrogen). Reverse transcription and rapid amplification of cDNA ends (RACE) was performed with the 5**′**-Full RACE Kit (TaKaRa, Dalian, China) in accordance with the manufacturer’s instructions. The final PCR product was extracted and purified from 2% agarose gel, cloned into pGEM-T Vector (Promega). Plasmid DNA from four different colonies was sequenced. Gene-specific primers used for 5′RACE experiments were listed in Supplementary Table S3.

### Cloning and sequence analysis of full-length SLC52A3 cDNA

SLC52A3 sequences were cloned by reverse transcriptase polymerase chain reaction (RT-PCR). Briefly, RNA was extracted from ESCC cell lines (EC8712) with TRIzol reagent (Invitrogen) and reverse transcribed using a reverse transcription system (TaKaRa). SLC52A3a and SLC52A3b coding sequences were amplified using the cloning primers listed in Supplementary Table S3, after which they were ligated into the pEASY-Blunt Simple Cloning Vector (TRANSGEN BIOTECH) and verified by complete sequencing.

### Construction of GFP-tagged SLC52A3a and SLC52A3b expression plasmids

The coding region of SLC52A3a and SLC52A3b was amplified and cloned into the *Hin*dIII and *Bam*HI sites of pEGFP-C1 vector (Clontech) to generate the GFP-SLC52A3a and GFP-SLC52A3b expression vector.

### Confocal immunofluorescence microscopy

The immunofluorescence staining was performed as described previously [26]. In brief, GFP-tagged SLC52A3a and SLC52A3b constructs were transfected into KYSE150 cells. At 48 h post-transfection, the cells were fixed in 4% paraformaldehyde for 10 min, after which they were rinsed with PBS and permeabilized in 0.1% Triton X-100 for 10 min. Nonspecific binding was blocked by incubating the cells with 5% normal donkey serum (Jackson ImmunoResearch) in PBS for 60 min. Subsequently, cells were incubated with antibodies against GFP (Santa Cruz Biotechnology), and then, cells were probed with the Alexa Fluor 488-conjugated AffiniPure donkey–anti-mouse secondary antibody (Jackson ImmunoResearch) and counterstained with DAPI (Sigma-Aldrich). Cells were analyzed using a Zeiss LSM880 confocal microscope (Zeiss). For common cell immunofluorescence, cells were seeded on a coverslip and incubated for 24 h. Pass by fixation and blocking, cells were incubated with primary SLC52A3 polyclonal antibodies (Abgent) in PBS for 4 h at room temperature and were probed with donkey–anti-rabbit Dylight 594 secondary antibodies (Jackson ImmunoResearch) in PBS for 1 h. To visualize the cell nucleus, cells were incubated with DAPI (Sigma-Aldrich) for 15 min and were imaged using an FV-1000 confocal microscopy (Olympus).

### shRNA lentivirus-mediated knockdown of SLC52A3

Recombinant lentivirus vectors pHBLV-U6-Luc-T2A-Puro harboring a short-hairpin RNA sequence targeting SLC52A3 (shSLC52A3-4#, shSLC52A3-6#, and control-shRNA) were produced by Hanbio Co., Ltd (Shanghai, China). To generate cells stably expressing shSLC52A3-4#, 6#, or the control-shRNA, lentivirus were used to infect KYSE180 and SHEEC cells, following the manufacturer’s instructions, when cells reached 40–50% confluence. Twenty-four hours later, cells were selected in medium containing 0.5 mg/mL puromycin (AMRESCO) for 20 days. After 4–5 passages in the presence of puromycin, the cultured cells were used for experiments.

### Adenovirus-mediated overexpression of SLC52A3a and SLC52A3b

To generate cells overexpressing SLC52A3a and SLC52A3b, the SLC52A3a and SLC52A3b CDS sequences were ligated into the pHBAd-MCMV-Luc Vector (Hanbio Co., Ltd., Shanghai, China). Then, KYSE150 and KYSE180 cells were infected with adenoviral vectors overexpressing SLC52A3a (Ad-SLC52A3a), SLC52A3b (Ad-SLC52A3b), or control adenovirus (Ad-Luc), at the MOI (multiplicity of infection) of 100 after 24 h of seeding. Thirty-six hours later, cells were passaged or harvested for further analysis.

### Cell proliferation assay

All cells were seeded in 96-well plates at 8000 cells per well. The CellTiter 96 aqueous nonradioactive cell proliferation assay was performed according to the manufacturer’s instructions (Promega) at 0, 24, 48, 72, and 96 h, respectively. 20 μL MTS was added to each well, and the plates were incubated for 2 h at 37 °C in a humidified, 5% CO_2_ atmosphere. MTS was bioreduced by the cells into a colored formazan product that displays absorbance at 490 nm. The absorbance was detected using a plate microplate reader (Multiskan MK3, Thermo). Raw data were normalized against those of the medium blank control.

### Colony formation assay

Colony formation assay was performed as described previously [27]. Briefly, transfected cells were plated at a density of 1000 cells per well in 6-well plates and incubated for 14 days at 37 °C with 5% CO_2_. After washing with 4 °C pre-cooled PBS, cultures were fixed with ice-cold methanol for 20 min and stained with hematoxylin for 15 min. Colonies were photographed and calculated using a FluorChem 8900 image analysis system (Alpha Innotech, Miami, FL, USA). Each experiment was performed in triplicate.

### Measurement of riboflavin

Both intra- or extra-cellular concentrations of riboflavin were measured by high-performance liquid chromatography (HPLC) as described previously [28]. Briefly, cells were plated at a density of 2 × 10^5^ cells per well in 6-well plates, and culture medium was collected after 0, 24, 48, and 72 h, respectively, while cells were collected after 72 h. The concentrations of riboflavin in the collected culture medium were measured directly by HPLC. Cells were lysed by ultrasonic wave and riboflavin concentrations were measured by HPLC.

### Reporter gene constructs

The human SLC52A3 5′-flanking region − 5076/− 2403 (translation initiation site for the SLC52A3 protein occurs at + 1) was generated by PCR using primers SLC52A3-5′FR-1F and SLC52A3-5′FR-1R (Supplementary Table S3). The amplified fragment from the genomic DNA of KYSE180 cells was digested with *Xho*I/*Bgl*II and inserted into the *Xho*I/*Bgl*II sites of pGL4.15[luc2P/Hygro] Vector (Promega), and the resulting plasmid was named pGL4 (− 5076/− 2403). Using the same methods, the luciferase reporter plasmids, pGL4 (− 3825/− 2403), pGL4 (− 3391/− 2403), pGL4 (− 2849/− 2403), pGL4 (− 3288/− 2403), pGL4 (− 3020/− 2403), pGL4 (− 2935/− 2403), pGL4 (− 2897/− 2403), pGL4 (− 2897/− 2495), pGL4 (− 2897/− 2583), pGL4 (− 2897/− 2672), pGL4 (− 2897/− 2743), and pGL4 (− 2897/− 2782) were generated. Different deletion fragments of SLC52A3-5′FR (− 3020/− 2672) and luciferase reporter plasmids, pGL4 (Δ− 2935/− 2897), pGL4 (Δ− 2897/− 2849), pGL4 (Δ− 2935/− 2849), and pGL4 (Δ− 2782/− 2743), were constructed by GENEWIZ (Suzhou, China).

### Dual-luciferase reporter assay

Dual-luciferase reporter assay was performed as described previously [29, 30]. Briefly, KYSE150 or HEK293T Cells were seeded into 96-well plates at the density of 1 × 10^4^ cells/well and cultured for 16–24 h until grown to 70–90% confluence. They were then co-transfected with a firefly luciferase expressing plasmid (1 μg), and a renilla luciferase-expressing plasmid (20 ng) (pRL-TK, Promega) as an internal control, using LIPOFECTAMINE 3000 Transfection Reagent (Thermo Fisher Scientific) in accordance with the manufacturer’s protocol. 48 h later, cells were harvested using passive lysis buffer (Promega). Luciferase activity was analyzed using the GloMax 96 Microplate Luminometer (Promega). Values for each group are expressed as the mean ± standard deviation (SD) for three separate experiments.

### Western blot analysis

Whole cell protein extracts collected from cells were prepared in 1× Laemmli Sample Buffer (Bio-Rad), nuclear cell protein extracts were isolated by NE-PER Nuclear and Cytoplasmic Extraction Reagents (Thermo Fisher Scientific), and membrane protein were isolated by Minute Plasma Membrane Protein Isolation Kit (Invent Biotechnologies, Inc.). The protein concentration was estimated by the Pierce 660 nm Protein Assay (Thermo). An equal amount of tissues lysates was electrophoresed on 10% polyacrylamide gel using standard methodology. Then, the lysates were transferred to PVDF membranes (Roche). The membranes were blocked in blocking buffer for 1 h followed by the addition of the primary antibody for 12 h at 4 °C. The membranes were then washed and incubated with a secondary antibody coupled to horseradish peroxidase for 1 h at room temperature. Antigen–antibody complexes were detected by Western blot luminol reagent (Santa Cruz Biotechnology). The primary antibodies used are as follows, anti-Ikkα, anti-Ikkβ, anti-phospho-Ikkα/β, anti-IκBα, anti-phospho-IκBα, anti-NF-κB p65 and anti-phospho-NF-κB p65 (ser536) (Cell Signaling Technology); anti-Rel-B, anti-NF-κB p50, anti-SLC52A3, anti-Integrin-α5, anti-Lamin A/C, and anti-β-actin (Santa Cruz Biotechnology); anti-GAPDH (Sigma). Image acquisition and quantitative analysis were carried out using the ChemiDoc XRS imaging system (Bio-Rad).

### RNA extraction and quantitative real-time PCR

Total RNA was extracted from cells with TRIzol reagent (Invitrogen) in accordance with the manufacturer’s instructions. Reverse transcription (RT) and real-time PCR were performed as described earlier [31]. Briefly, complementary DNA (cDNA) was generated from 1 μg total RNA in a final volume of 20 μL with Reverse Transcription System (TaKaRa). For the normalization of the amount of each transcript, the housekeeping gene ACTB (β-actin) was used as the internal control. The quantitative RT-PCR assay was carried out with the 7500 Real-Time PCR Systems system (Applied Biosystems) using SYBR Premix Ex Taq (TaKaRa) in accordance with the manufacturer’s instructions. Primers for quantitative RT-PCR are shown in Supplementary Table S3. The comparative delta–delta Ct (2^−ΔΔCT^) method was used to calculate relative expression levels [32].

### Chromatin immunoprecipitation

Chromatin immunoprecipitation (ChIP) analysis was performed using EZ-Magna ChIP A/G Chromatin Immunoprecipitation kit (Millipore) in accordance with the manufacturer’s instructions. Briefly, KYSE150 cells were seeded into 100 mm cell culture dishes at the density of 1 × 10^5^ cells per dish. Following formaldehyde crosslinking, cell lysate was sonicated on wet ice and then centrifuged to precipitate the debris. Input sample (5%) was collected before immunoprecipitation. Supernatant containing cross-linked chromatin was incubated overnight at 4 °C with 1 μg of specific antibodies, including anti-STAT3 (Santa Cruz), anti-NF-κB p65 (Santa Cruz), anti-Rel-B (Santa Cruz), anti-RNA polymerase II (Millipore), and normal mouse IgG (Millipore). After incubation, samples were subjected to DNA purification. Finally, purified DNA was analyzed by real-time PCR (qPCR) using *SLC52A3* 5′-flanking region primers (Supplementary Table S3), according to procedures described previously [27].

### Electrophoretic mobility shift assay

LightShift Chemiluminescent Electrophoretic mobility shift assay (EMSA) kit and Chemiluminescent Nucleic Acid Detection Module were purchased from Thermo Fisher Scientific. Purified biotin-labeled probes were synthesized from Sangon Biotech, Shanghai, China. The probe sequences were in the Supplementary Table S3. Nuclear extracts of KYSE150 cells were isolated by NE-PER Nuclear and Cytoplasmic Extraction Reagents (Thermo Fisher Scientific). For binding assays, 2 μg nuclear extracts were incubated with 20 fmol probes at room temperature for 25 min in 20 μL reaction buffers. Electrophoreses, transferring to a nylon membrane and crosslinking at high temperature (120 °C), were performed in accordance with the manufacturer’s protocol. To confirm the specificity of the binding between transcription factors and the probes, antibodies anti-STAT3, anti-NF-κB p65, anti-Rel-B (Santa Cruz), and 2 μg nuclear extracts were incubated for 10 min at 4 °C.

### Statistical analysis

Data analysis was performed using SPSS 16.0 software. A two-tailed independent sample *t* test was used to determine the significance of differences between groups. Differences were considered statistically significant at *P* < 0.05 (*), *P* < 0.01 (**), and *P* < 0.01 (***). Data are plotted as mean ± SD.

## Results

### SLC52A3 protein was upregulated during the stepwise development of ESCC

**Fig. 1:**
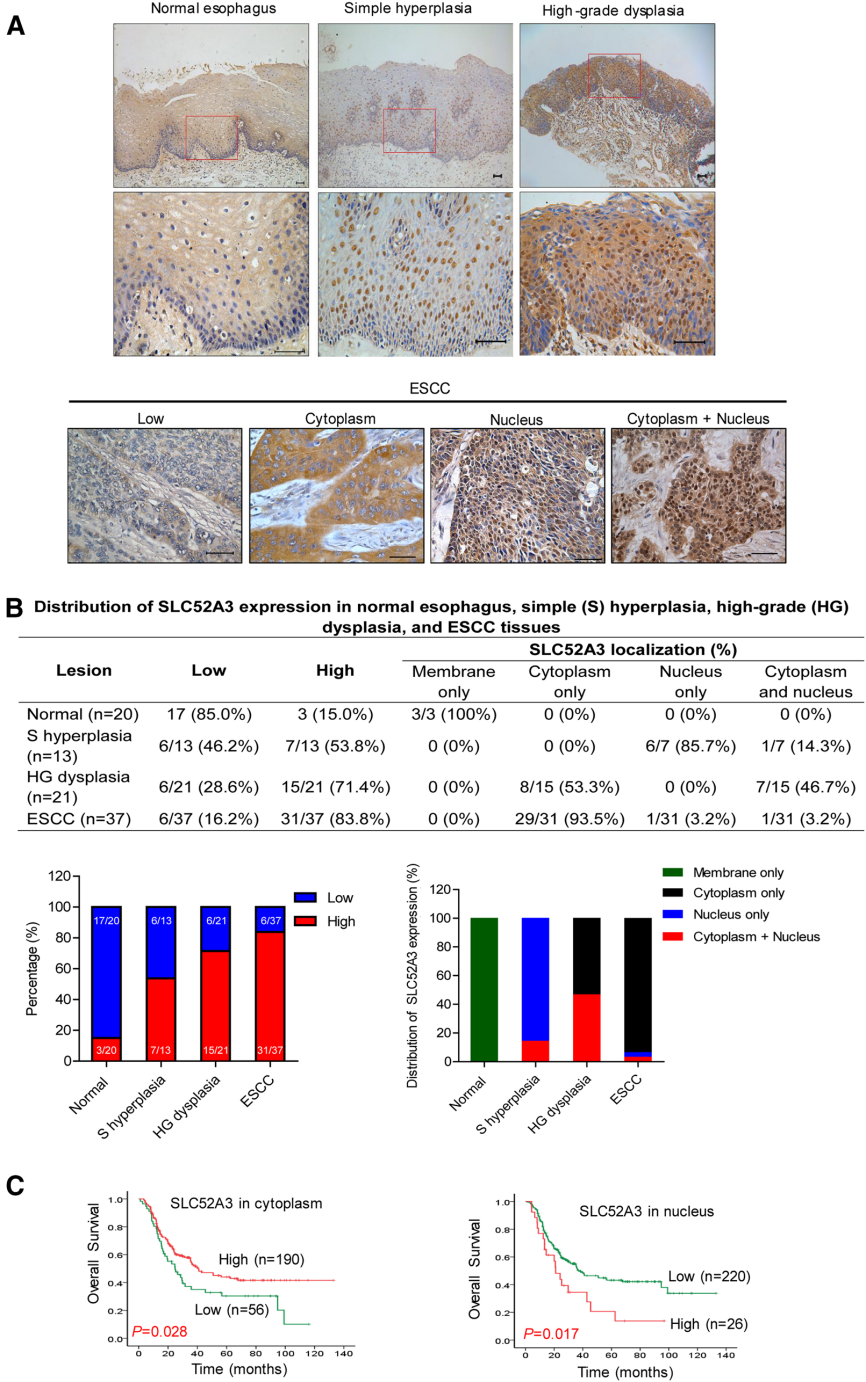
Expression and prognostic significance of SLC52A3 in normal esophageal epithelium and ESCC. **a** Immunohistochemical staining of SLC52A3 during the process of transformation and development of ESCC. Scale bars, 50 μm. **b** Distribution of SLC52A3 expression in normal esophagus, simple (S) hyperplasia, high-grade (HG) dysplasia, and ESCC tissues. **c** Overall survivals of 246 patients with ESCC versus total SLC52A3 status. Higher SLC52A3 immunoreactivity in membrane and cytoplasm was associated with longer survival time of ESCC patients (*P* = 0.028). Higher SLC52A3 immunoreactivity in nucleus was associated with poor prognosis of ESCC patients (*P* = 0.017)

The stepwise development from normal squamous epithelium to ESCC can be characterized by histologic analysis, from simple hyperplasia to low-grade and high-grade dysplasia, carcinoma in situ, and finally to invasive carcinoma. To investigate the alterations of SLC52A3 protein expression and their clinical significance during ESCC development, we first procured 39 ESCC individuals with most of whom having matched normal esophagus epithelium, simple hyperplasia, high-grade dysplasia, as well as ESCC samples. By immunohistochemical (IHC) staining, distinct SLC52A3 expression patterns were noted during stepwise ESCC developmental process. Specifically, normal esophagus epithelial cells were weakly positive for SLC52A3 signals. In contrast, distinctive nucleic staining of SLC52A3 was observed in simple hyperplasia of the esophagus. Notably, strong cytoplasmic and nucleic staining of SLC52A3 was detected in high-grade dysplasia, while diffuse cytoplasmic staining was observed in most of ESCC tissues (Fig. 1a, b). These data strongly imply the potential biological significance of SLC52A3 in ESCC. More importantly, these approaches identify SLC52A3 as a predictive biomarker for the development of ESCC.

To further explore the clinical significance of the expression of SLC52A3 protein, we next performed IHC in a large independent cohort with 246 ESCC patients. Importantly, survival analysis revealed that SLC52A3 protein expression was a prognostic factor in ESCC. Specifically, stronger immunoreactivity of SLC52A3 in the membrane and cytoplasm was associated with a better survival (*P* = 0.028, Fig. 1c). In contrast, higher expression of SLC52A3 in nucleus was significantly associated with poor prognosis (*P* = 0.017, Fig. 1c).

### Identification of a novel SLC52A3 isoform in ESCC

**Fig. 2:**
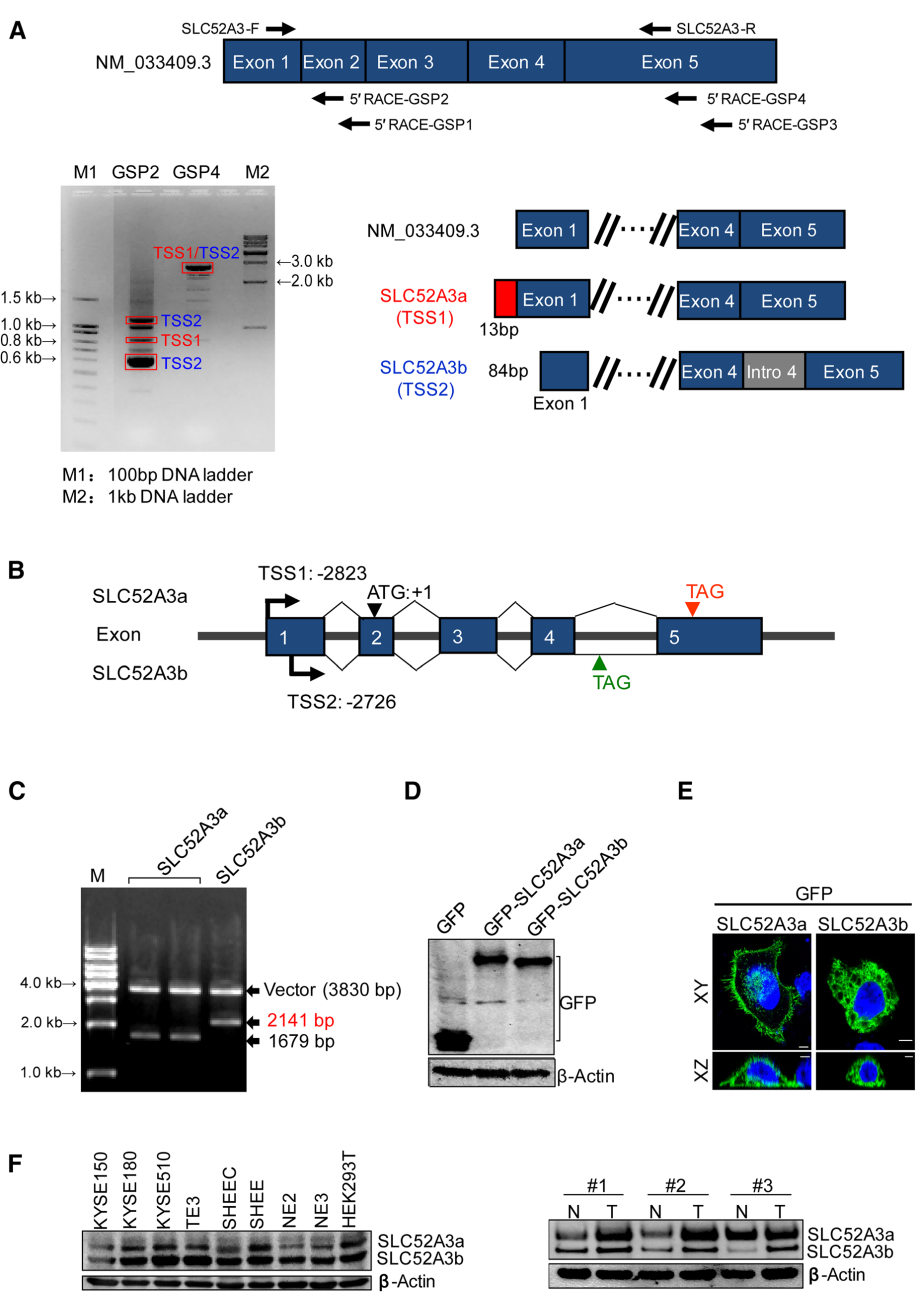
Determination of the transcription start sites for the *SLC52A3* gene and identification of the SLC52A3 isoforms. **a** Identification of the transcription start sites (TSS) of the SLC52A3 transcripts using 5′RACE analysis in KYSE150 cells. 5′RACE experiments were repeated three times and a representative gel image is shown. Relative positioning of the oligonucleotide primers used for 5′RACE amplification (up); amplification products by agarose electrophoresis and schematic of sequencing results (down). **b** mRNA schematic of SLC52A3a and SLC52A3b. **c** Full-length cDNA cloning and the double restriction enzyme digestion (*Eco*RI and *Bam*HI) of SLC52A3a and SLC52A3b. **d** Western blot analysis of GFP-tagged SLC52A3a and SLC52A3b transfected into KYSE150 cells. **e** Immunofluorescence analysis of SLC52A3a and SLC52A3b in KYSE150 cells. The GFP-tagged SLC52A3a and SLC52A3b were labeled with Alexa Fluor 488 (*green*) and nuclei were counterstained with DAPI (*blue*). **f** SLC52A3 was analyzed via western blot in cell lines and esophageal squamous cell carcinoma (ESCC) tissues, and β-actin was used as the internal control

Given the diversity of SLC52A3 expression in the ESCC tissue, we speculated that different transcription variants of SLC52A3 might exist. To test this, 5′RACE experiments were performed using total RNA extracted from TE3 cells (an ESCC line). The results showed that *SLC52A3* gene had two different transcription initiation sites (TSSs), namely, TSS1 and TSS2 (Fig. 2a). These two TSSs were located at 2823 and 2726 bp upstream from translational start codon, respectively. Notably, TSS2 contained intron 4. Compared with RefSeq databases, TSS1-initiated transcript corresponded to SLC52A3 mRNA (NM_033409.3), which here and after we named SLC52A3a. Thus, these results identified TSS2-initiated transcript as a novel alternative splicing isoform, which here and after we named SLC52A3b (Fig. 2b). The novel SLC52A3b isoform was confirmed by double restriction enzyme digestion (Fig. 2c). Sequence analysis also verified that SLC52A3b retained the 4th intron and premature termination. Data from the sequence analysis have been submitted to the GenBank database (SLC52A3a, GenBank accession No. KY978478; SLC52A3b, GenBank accession No. KY978479; also available in Supplementary Figure S1 and Figure S2).

This new SLC52A3b variant encodes a protein of 415 amino acids with predicted molecular mass of 45 kDa. We next constructed GFP-tagged expression plasmids for SLC52A3a and SLC52A3b (Fig. 2d). Confocal immunofluorescence microscopy showed that the majority of SLC52A3a expressed in cell membrane and nucleus, while SLC52A3b localized in cell cytoplasm (Fig. 2e). We next measured endogenous SLC52A3 proteins in ESCC cell lines and primary tissues by Western blotting, and again, both isoforms were evident in ESCC cell lines (Fig. 2f). Notably, higher protein levels of SLC52A3a and SLC52A3b were also observed in ESCC tumor samples compared with matched normal tissues, in agreement with our earlier IHC results (Fig. 2f).

### High SLC52A3a nucleic expression correlates with poor prognosis in ESCC patients

**Fig. 3:**
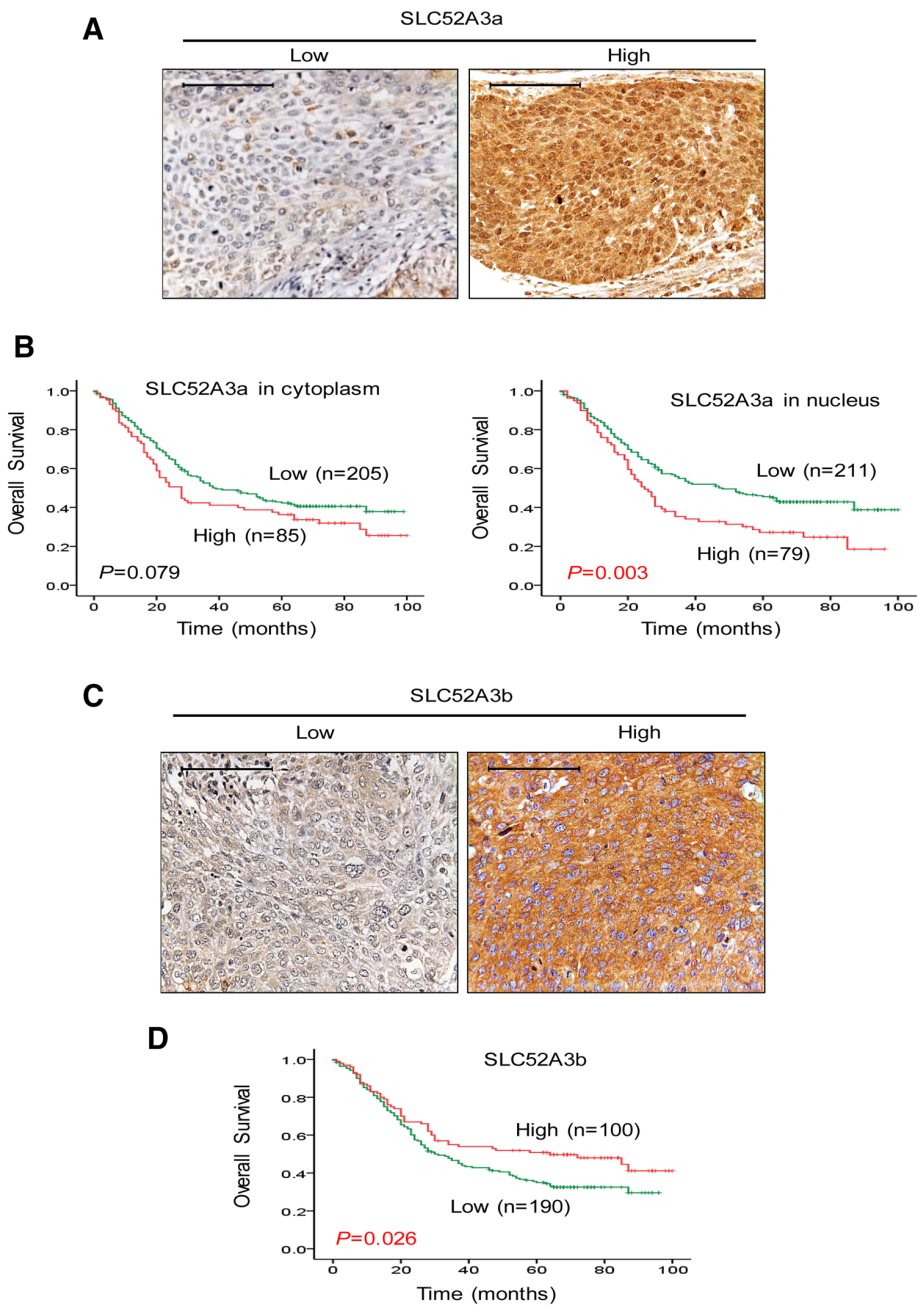
Expression and prognostic significance of SLC52A3a and SLC52A3b in ESCC. **a** SLC52A3a expression was detected by IHC in 290 ESCC samples. Cytoplasmic and nuclear staining of SLC52A3a were observed. Scale bars, 50 μm. **b** SLC52A3a expression in cytoplasmic was not associated with survival time of ESCC patients (*P* = 0.079). Higher SLC52A3a immunoreactivity in nucleus was associated with poor prognosis of ESCC patients (*P* = 0.003). **c** SLC52A3b expression was detected by IHC in 290 ESCC samples. Cytoplasmic staining of SLC52A3b was observed. Scale bars, 50 μm. **d** Higher SLC52A3b immunoreactivity in cytoplasmic was associated with poor prognosis of ESCC patients (*P* = 0.026)

To further explore the clinical significance of expression of SLC52A3a and SLC52A3a proteins, we first generated antibodies specifically recognizing different isoforms and confirmed their stringent specificity using recombinant SLC52A3 polypeptide by western blot (See Method). We next performed IHC in another large independent cohort with 290 ESCC patients. Importantly, stronger immunoreactivity of SLC52A3a in nucleus was significantly associated with poor prognosis of ESCC patients (*P* = 0.003), while its expression in cytoplasmic was not prognostic (*P* = 0.079) (Fig. 3a, b). On the contrary, heightened expression of SLC52A3b (cytoplasm) was significantly associated with favorable prognosis of ESCC patients (*P* = 0.026) (Fig. 3c, d). These data strongly suggest distinct functions of the two isoforms in the biology of ESCC cells.

### Functional roles of SLC52A3 in ESCC

**Fig. 4:**
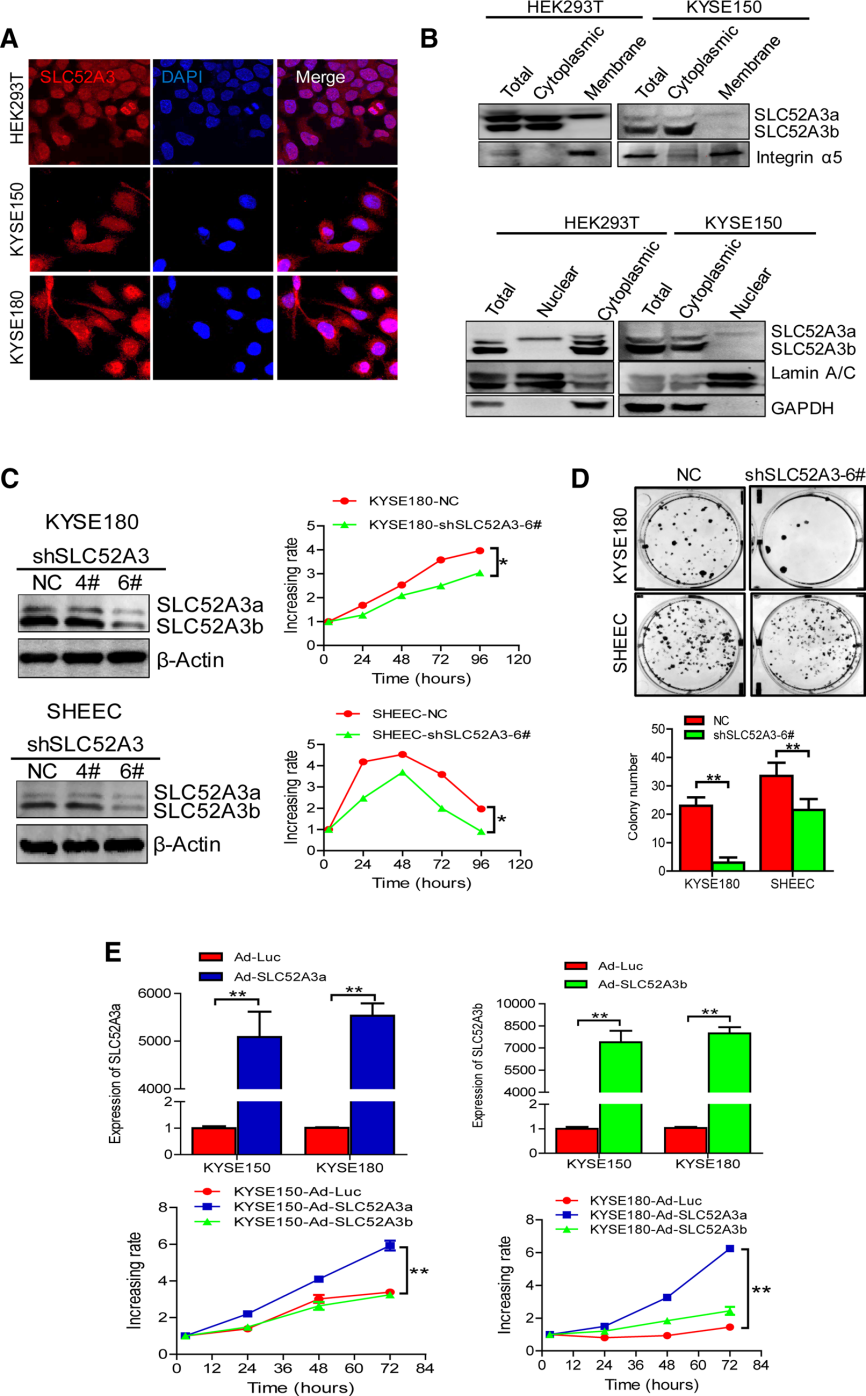
Localization and function of SLC52A3 in human ESCC cells. **a** Immunofluorescence analysis of SLC52A3 in HEK293T, KYSE150, and KYSE180 cells. The target protein and nuclei were labeled, respectively, with Dylight594 (red) and DAPI (blue). **b** Sub-cellular fractionations of HEK293T and KYSE150 cells were made as described in Materials and Methods. The fractions were analyzed with anti-SLC52A3, anti-Integrin α5 as membrane, anti-Lamin A/C as nuclear, and anti-GAPDH as cytoplasmic marker antibody. Shown are representative data of two independent experiments. **c** SLC52A3 ablation in KYSE180 and SHEEC cells was confirmed by Western blot analysis (left). MTS assay of KYSE180 and SHEEC cells after SLC52A3 knockdown (right). Experiments were repeated three times with similar results. **d** shRNA lentivirus-mediated SLC52A3 knockdown KYSE180 and SHEEC cells showed a significantly reduced colony formation compared with control cells. Representative pictures (top) and quantitative analyses (bottom) of colony numbers. Data show representative colony formation assay for each condition performed in triplicate ± SD, for three independent experiments. **e** KYSE150 and KYSE180 cells were overexpression with SLC52A3a or SLC52A3b by adenovirus-mediated. The efficiency of SLC52A3a or SLC52A3b overexpression was evaluated using real-time RT-PCR (top). MTS assay of KYSE150 and KYSE180 cells after SLC52A3a or SLC52A3b overexpression (bottom). Experiments were repeated three times with similar results. Error bars indicate SD. *P < 0.05, **P < 0.01 based on Student’s *t* tests

We examined the sub-cellular localization of SLC52A3 in HEK293T, KYSE150, and KYSE180 cells. Confocal microscopy showed that SLC52A3 was detectable in all sub-cellular compartments, including cell membrane, cytoplasm, as well as nucleus (Fig. 4a). Next, upon cell fractionation, western blotting verified that SLC52A3a expressed in cell membrane, cytoplasm, and nucleus, while the majority of SLC52A3b expressed in cell cytoplasm (Fig. 4b). These results were concordance with the findings from IHC assays in primary ESCC tissues.

We next sought to investigate the biological functions of SLC52A3 using ESCC cell line models. First, we determined the transport capacity of riboflavin by either SLC52A3a or SLC52A3b in KYSE150 and KYSE510 cells by measuring both riboflavin consumption in cell culture medium and intracellular riboflavin concentration using high-performance liquid chromatography (HPLC). Importantly, our results showed that cells expressing SLC52A3a exhibited faster riboflavin consumption and maintained higher intracellular concentration of riboflavin compared to control cells. In contrast, expression of SLC52A3b did not cause any alterations in either riboflavin consumption or intracellular riboflavin concentration (Supplementary Figure S3), suggesting that SLC52A3a has higher capacity in transporting riboflavin than SLC52A3b. Importantly, shRNA-mediated knockdown of SLC52A3 (shSLC52A3-6#) markedly decreased the proliferation of both KYSE180 and SHEEC cells (Fig. 4c). ESCC colony formation was also potently inhibited upon silencing of SLC52A3 (Fig. 4d). We next ectopically expressed either isoforms, and noted that overexpression of SLC52A3a significantly increased the proliferation of both KYSE150 and KYSE180 cells. In contrast, overexpression of SLC52A3b did not produce the same effect. These data together suggest that isoform SLC52A3a, but not SLC52A3b, promotes the malignant phenotype of ESCC cells (Fig. 4e).

### Identification of transcriptional regulatory elements in *SLC52A3* 5′-flanking regions

**Fig. 5:**
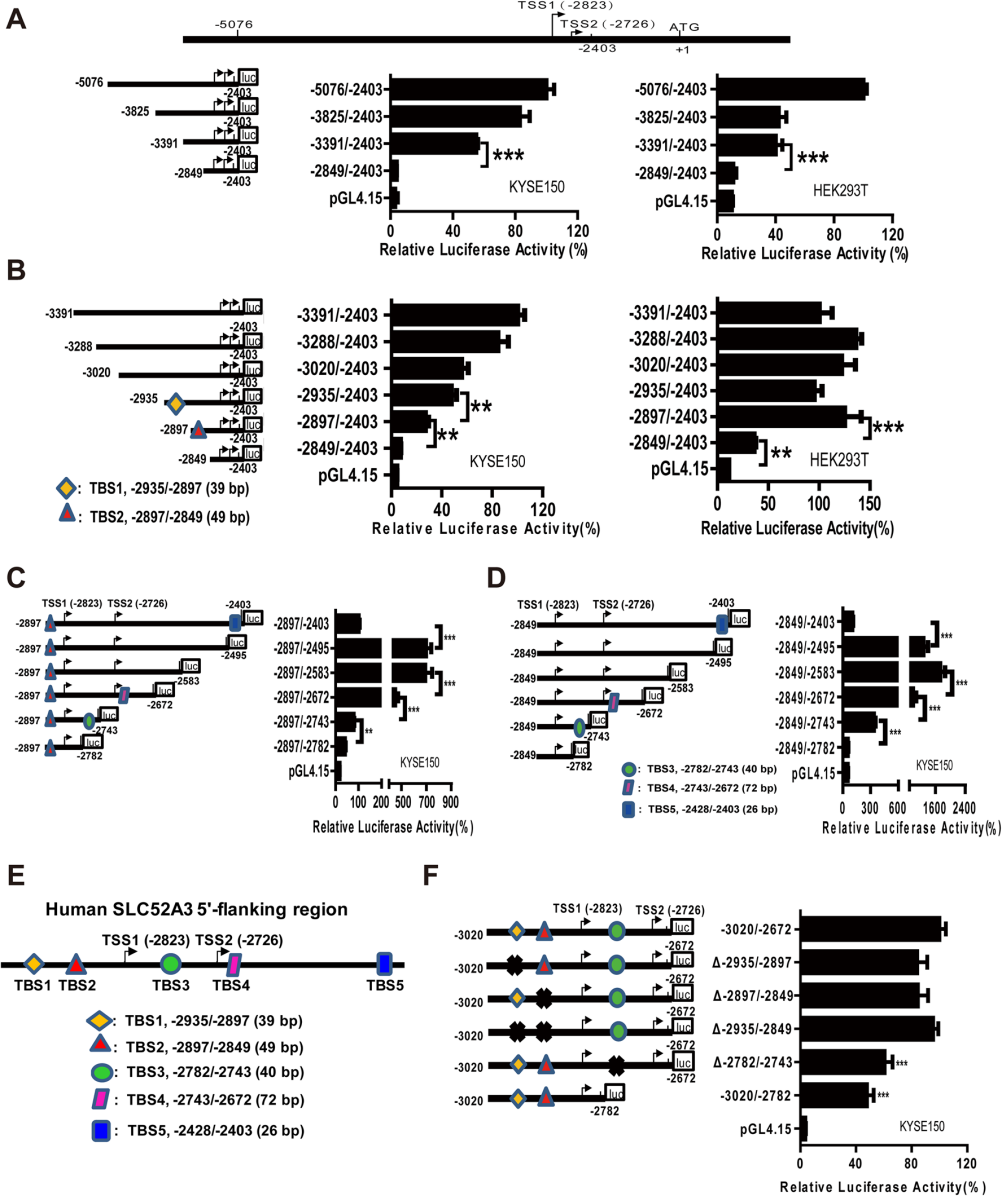
Transcriptional regulatory region of the human *SLC52A3* 5′-flanking region − 5076/− 2403. Localization of the transcriptional regulatory region of human *SLC52A3* by 5′-deletion analysis **a**, **b** Schematic representation of the *SLC52A3* 5′-flanking region constructs used for transient transfections is shown in the left. 5′-Deletion constructs were co-transfected with pRL-TK into KYSE150 and HEK293T cells. Luciferase activity (right) was normalized to Renilla luciferase activity and then shown relative to that of cells transfected with pGL4-hS (− 5076/− 2403) (**a**) or pGL4-hS (− 3391/− 2403) (**b**), which were set to 100%. Localization of the transcriptional regulatory region of human *SLC52A3* by 3′-deletion analysis (**c**, **d**) and fragments deletion analysis (**f**) in KYSE150 cells. **e** Schematic of SLC52A3 5′-flanking region transcriptional regulatory elements (TBS1-5). Luciferase activity was normalized to Renilla luciferase activity and then shown relative to that of cells transfected with pGL4 (− 2897/− 2403) (**c**), pGL4 (− 2849/− 2403) (**d**) or pGL4 (− 3020/− 2672) (**f**), which were set to 100%. Each value represents the mean ± SD. The data are representative of at least two independent experiments. Transfections were carried out in six times repeated for each experiment (*n* = 6). **P < 0.01; ***P < 0.001

We next probed the mechanisms underlying the upregulation of SLC52A3 expression in ESCC. To identify its transcriptional regulatory elements, a series of *SLC52A3* 5′-flanking regions (spanning − 5076/− 2403 upstream of translational starting codon) were cloned into reporter gene constructs. The − 5076/− 2403 region of *SLC52A3* exhibited maximum luciferase activity, and sequence deletion from nt − 3391 to nt − 2849 led to an ~ 80% reduction in luciferase activity (Fig. 5a). We thus continued to fine-map this region by further serial deletions. Importantly, both deletions of − 2935/− 2897 and − 2897/− 2849 markedly decreased the reporter activity in KYSE150 cells, while only the deletion of − 2897/− 2849 strongly decreased the activity in HEK293T cells (Fig. 5b). These data suggest that region − 2897/− 2849 operates as the basic (nontissue-specific) regulatory element of *SLC52A3*, and region − 2935/− 2897 might be an ESCC specific regulatory element.

To further investigate the transcriptional regulatory elements of *SLC52A3*, a series of 5′-flanking regions spanning − 3020/− 2403 were studies by progressive 3′-deletions, which initially increased the reporter activity and then followed by decreases. Specifically, deletion from nt − 2403 to nt − 2495 caused a 16-fold increase in luciferase activity, whereas deletion from − 2583/− 2672, − 2672/− 2743, and − 2743/− 2782 markedly decreased the activity (Fig. 5c, d). This result indicated that 5′-flanking regions of *SLC52A3* gene contained at least five transcriptional regulatory elements, namely TBS1–TBS5, as illustrated in Fig. 5e.

Further progressive deletions of either TBS1, TBS2, or both TBS1/TBS2 did not alter the reporter activity. However, the deletion of TBS3 sharply and significantly reduced reporter activity (Fig. 5f), suggesting that a key cis-acting element exists in this segment (− 2782/− 2743) which regulates *SLC52A3* transcription in ESCC cells.

### NF-κB p65/Rel-B factor binds to the NF-κB-binding site within TBS3 and activates SLC52A3 transcription

**Fig. 6:**
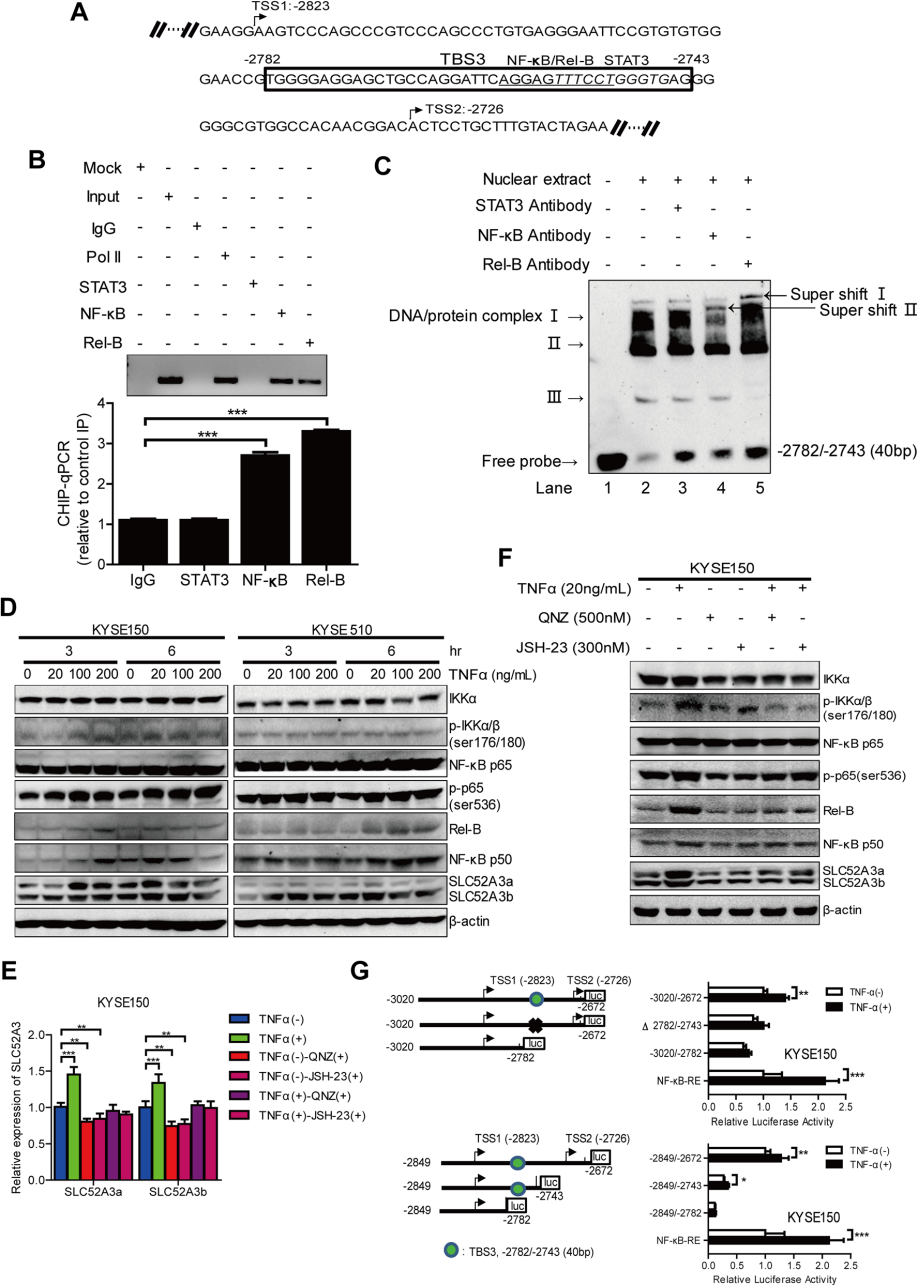
Transcription factors NF-κB p65/Rel-B binds to the NF-κB-binding site within the − 2782/− 2743 fragment of the *SLC52A3* 5′-flanking region and TNFα-triggered NF-κB-cell signaling upregulates transcriptional expression of human SLC52A3. **a** Bioinformatics analysis of regulatory elements in *SLC52A3* 5′-flanking region. Transcription factors prediction of 5′-flanking region TBS3 (− 2782/− 2743) by Alibaba 2.1 and JASPAR. NF-κB p65/Rel-B-binding site: underline; STAT3 binding site: italic. **b** ChIP-qPCR analysis of STAT3, NF-κB p65 and Rel-B in immunoprecipitated DNA fragments on *SLC52A3* 5′-flanking region. Semi-quantitative PCR agarose gel electrophoresis (*left*) and quantitative PCR (*right*) results were showed. anti-RNA polymerase II was used as a positive control and normal mouse IgG was used as a negative control. Data are normalized to total input DNA and expressed as mean ± SD of three independent samples. ***P < 0.001. **c** EMSA assay of the nuclear extract prepared from KYSE150 cells bound to the sequence of − 2849/− 2743 fragment within the *SLC52A3* 5′-flanking region. Lane 1 was loaded only with the nuclear extract; lane 2 was loaded with the biotin-labeled probe and nuclear extract; lanes 3, 4, 5 were loaded with the biotin-labeled probe, nuclear extract, and antibody (anti-STAT3, anti-NF-κB p65 or anti-Rel-B). **d** Expression of NF-κB-cell signaling proteins and SLC52A3 was evaluated using western blot analysis in various concentrations and times TNFα-treated KYSE150 and KYSE510 cells. **e** KYSE150 cells were pretreated with QNZ (500 nM) or JSH-23 (300 nM) for 24 h, followed by TNFα (20 ng/mL) treatment for 6 h. The expression of SLC52A3 mRNA was evaluated using qRT-PCR. The data are representative of three independent experiments. **f** KYSE150 cells were treated as described above, and cell lysis was quantified and subjected to western blot to detect NF-κB-cell signaling proteins and SLC52A3 activation. Blots are representative of three independent experiments. **g** Dual-luciferase reporter assay *SLC52A3* 5′-flanking region (− 3020/− 2672) (up) and (− 2849/− 2672) (down) activity by 3′-deletion or fragments deletion in KYSE150 cells with or without TNFα treated, pGL4.32[luc2P/NF-κB-RE] (Promega) as the positive control. The data are representative of at least two independent experiments. Transfections were carried out in six times repeated for each experiment (*n* = 6). *P < 0.05; **P < 0.01; ***P < 0.001

The above data prompted us to further investigate transcription factor-binding sites within TBS3. Thus, we subjected this genomic region to different motif analysis methods (Fig. 6a), and identified binding motifs for NF-κB p65/Rel-B (− 2760/− 2750, underlined) and STAT3 (− 2755/− 2745, italic). We then performed ChIP analysis to test the prediction. Immunoprecipitated chromosomal DNA was subjected to semi-quantitative PCR agarose gel electrophoresis and qPCR analysis (anti-RNA polymerase II and normal mouse IgG were used as a positive control and negative control, respectively). Importantly, NF-κB p65 and Rel-B, indeed, bound to this region, whereas STAT3 did not (Fig. 6b). To assess the specificity of NF-κB p65/Rel-B binding, nuclear extracts of KYSE150 cells were incubated with a biotinylated oligonucleotide containing the NF-κB p65/Rel-B and STAT3 motif sequences within TBS3. Signals in EMSA results showed that, compared with the nuclear extract and probe control (Fig. 6c, lane 2), either anti-NF-κB p65 or anti-Rel-B antibody generated super-shift complexes, which was not formed by anti-STAT3 antibody (Fig. 6c, lane 3, 4 and 5). These results together confirmed that NF-κB p65 and Rel-B bound to NF-κB-binding motif (from nt − 2760 to nt − 2750) within TBS3 element of *SLC52A3* 5′-flanking region.

NF-κB family members modulate the transcription of a number of genes that regulate inflammation, apoptosis, and tumorigenesis [33–36]. Tumor necrosis factor α (TNFα) is among the best-characterized activators upstream of NF-κB signaling pathway [37]. To explore whether NF-κB p65/Rel-B regulate the transcription of SLC52A3, KYSE150, and KYSE510 cell lines were treated with various concentrations of TNFα. As expected, TNFα stimulation increased NF-κB activity, as evident by the enhanced phosphorylation of IKKα/β, as well as increased expression of Rel-B and p50. Notably, activation of NF-κB pathway resulted in strong upregulation of SLC52A3 expression, particularly SLC52A3a (Fig. 6d). On the other hand, treatment of NF-κB inhibitors QNZ or JSH-23 substantially decreased the expression levels of SLC52A3a and SLC52A3b (Fig. 6e). NF-κB signaling pathway-related proteins and SLC52A3, specially SLC52A3a, were also decreased in KYSE150 cells (Fig. 6f).

To further confirm the transcriptional regulation of NF-κB on TBS3 segment, we performed progressive deletions for reporter luciferase assay under the treatment of TNFα. Importantly, TNFα stimulated the reporter activity of all constructs except for the one without NF-κB-binding sequences (Fig. 6g, upper panel). Similar results were obtained by serial 3′ deletions (Fig. 6g, lower panel). These results together suggest that NF-κB signaling activates SLC52A3 transcription through direct binding to its canonical sequences within *SLC52A3* 5′-flanking region.

## Discussion

Recent results demonstrated profound epigenomic dysregulation of ESCC transcriptomes [38–41]. In the present study, we found that SLC52A3 was transcriptionally upregulated in both esophagus dysplasia and ESCC, and high nucleus expression of SLC52A3 was correlated with poor prognosis. Importantly, SLC52A3 promoted cell proliferation and colony formation in ESCC. Our results further showed that SLC52A3 had two transcript variants that differ in their transcriptional start sites (TSS) and encoded two different proteins. The previous studies reported that intron retention may be ascribed to the dysfunction of splicing factors or weaker splice sites [42, 43]. However, whether alternative transcription start site and splicing are functionally linked remains unclear. Further investigations are thus warranted in the future.

A previous study reported the minimal promoter of *SLC52A3* [44], which were in agreement with our data. We here, however, identified an additional TSS of *SLC52A3* using RACE. Luciferase reporter assay revealed that 5′-flanking regions of *SLC52A3* contain at least five transcriptional regulatory elements (TBS1–TBS5). Furthermore, our results revealed and validated a functional-binding site for NF-κB p65/Rel-B within TBS3. Interestingly, the previous GWAS results showed that an SNP locus rs13042395 (C>T), located − 8093 nt upstream of *SLC52A3* TSS, was associated with increased ESCC risk [45, 46]. A study demonstrated that the CC genotype of this SNP conferred stronger transcription activity for *SLC52A3* [47]. Together with our results, these findings suggest a complex regulatory mechanism controlling SLC52A3 transcription in ESCC.

**Fig. 7:**
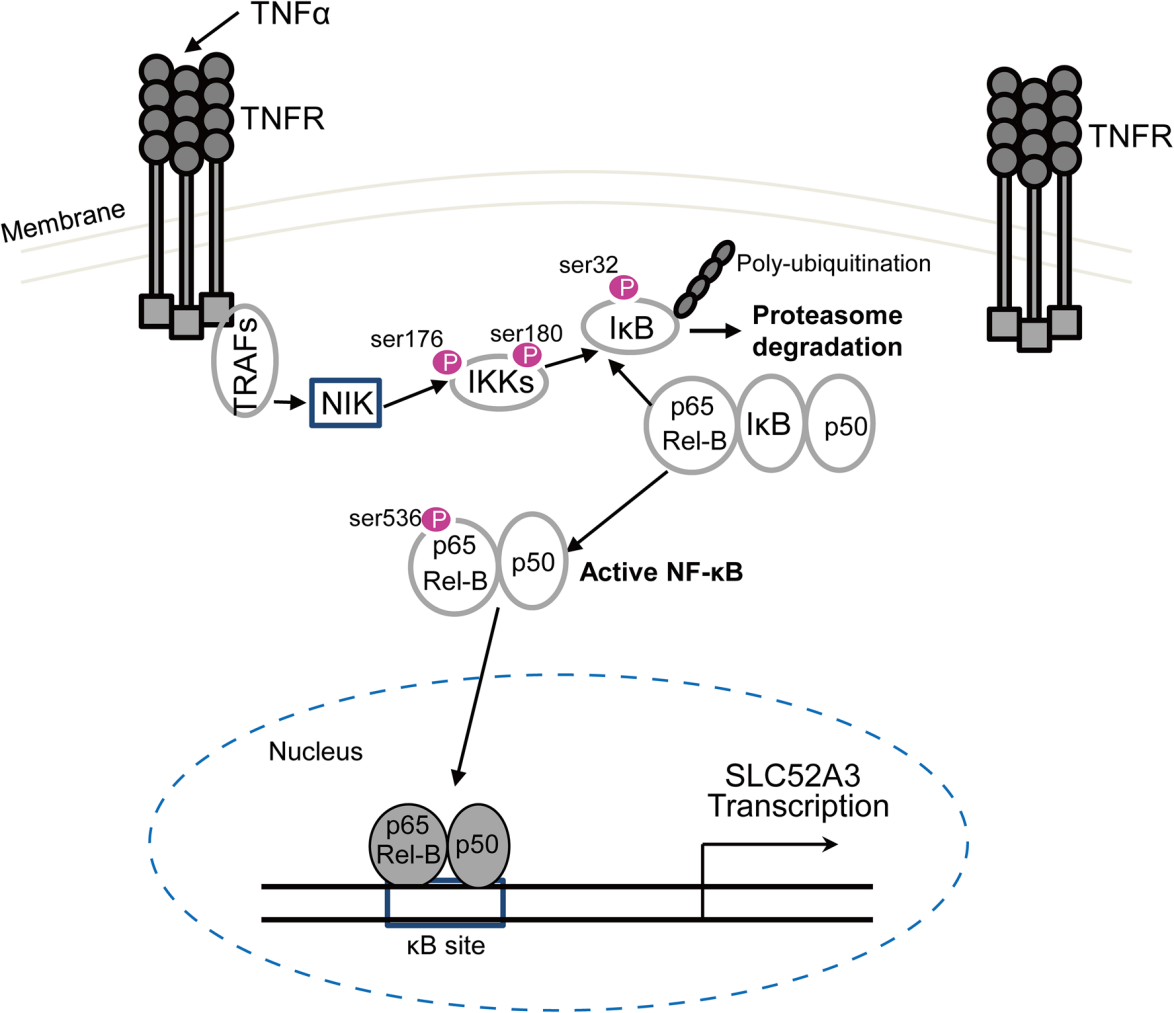
Schematic model of the NF-κB pathway in the regulation of the human SLC52A3 transactivation. Environmental stimuli that activate NF-κB p65/Rel-B cause phosphorylation of IκB, which is followed by its ubiquitination and subsequent degradation. This results in the exposure of the nuclear localization signals (NLS) on NF-κB p65/Rel-B subunits and the subsequent translocation of the molecule to the nucleus. In the nucleus, NF-κB p65/Rel-B bind to their consensus sequence in the human SLC52A3, resulting in SLC52A3 transcriptional activation

Recently, multiple studies highlighted the pro-inflammatory effects of low concentration of riboflavin [9, 48–50], presumably resulting in the release of a number of cytokines, which in turn causes the activation of NF-κB. NF-κB p65 is upregulated in ESCC tissues, and its hyper-activation plays a role in the occurrence, development and metastasis of ESCC [51–53]. The present study found that TNFα induced overexpression of SLC52A3 in ESCC cells by activating NF-κB signaling (Fig. 7). We speculate that the riboflavin deficiency in ESCC cells increases TNFα and activates NF-κB, which increases the expression of SLC52A3, providing a mechanistic explanation for the negative feedback regulatory mechanisms maintaining riboflavin homeostasis inferred from the previous studies [9, 54].

In conclusion, our findings establish the clinical significance of SLC52A3 expression in the pathogenesis of ESCC, and highlight both the predictive and prognostic values of this pro-growth protein. We further identified isoform-specific functions of SLC52A3, in which SLC52A3a, but not SLC52A3b, promotes the malignant phenotypes of ESCC cells. Mechanistically, we reveal a direct transcription regulation of NF-κB p65/Rel-B on the expression of SLC52A3, providing important insights into the tumorigenesis and progression of ESCC.

## Electronic supplementary material

Below is the link to the electronic supplementary material.

Supplementary material 1 (DOCX 173 kb)

## Abbreviations

ChIP: Chromatin immunoprecipitation
EMSA: Electrophoretic mobility shift assay
ESCC: Esophageal squamous cell carcinoma
FR: Flanking regions
RACE: Rapid amplification of cDNA ends
RT-PCR: Reverse transcriptase polymerase chain reaction
TRADD: TNF receptor death domain
TSS: Transcriptional start site
PBS: Phosphate buffer saline
