# Supplementary material for: SLC52A3 expression is activated by NF-κB p65/Rel-B and serves as a prognostic biomarker in esophageal cancer
Source: Cell Mol Life Sci. 2018 Feb 10;75(14):2643–61. doi: 10.1007/s00018-018-2757-4 (PMC6003972; doi:10.1007/s00018-018-2757-4)
Supplement: Supplementary file 1 — Supplementary material 1 (DOCX 173 kb) [file 18_2018_2757_MOESM1_ESM.docx]

SLC52A3 expression is activated by NF-κB p65/Rel-B and serves as a prognostic biomarker in esophageal cancer

Lin Long^1,2,3^, Xiao-Xiao Pang^2,3^, Fei Lei^1,2^, Jia-Sheng Zhang^1,2^, Wei Wang^1,2^, Lian-Di Liao^2,3^, Xiu-E Xu^2,3^, Jian-Zhong He^2,3^, Jian-Yi Wu^1,2^, Zhi-Yong Wu^4^, Li-Dong Wang^5^, De-Chen Lin^6*^, En-Min Li^1,2*^, Li-Yan Xu^2,3*^

**Supplementary Files:**

**Supplementary Figure S1.** Homo sapiens solute carrier family 52 member 3a (SLC52A3a), mRNA sequence.

**Supplementary Figure S2.** Homo sapiens solute carrier family 52 member 3b (SLC52A3b), mRNA sequence.

**Supplementary Figure S3.** Concentrations of riboflavin in culture medium and intracellular were quantified by HPLC.

**Supplementary Table S1.** The clinical characteristics and survival information of the patients for the immunohistochemical staining (Shantou Central Hospital from 1987 to 1997, 246 cases).

**Supplementary Table S2.** The clinicopathological characteristics of generation dataset of patients with ESCC (Shantou Central Hospital from 2007 to 2014, 290 cases).

**Supplementary Table S3.** PCR primers used in this study.

**Supplementary Figure S1**

**Homo sapiens solute carrier family 52 member 3a (SLC52A3a), mRNA**

GenBank accession [No. KY978478](https://www.ncbi.nlm.nih.gov/nuccore/KY978478).

>SEQ1 [organism=Homo sapiens] Homo sapiens solute carrier family 52 member 3 (SLC52A3), transcript variant 1, mRNA, SLC52A3a

>Feature SEQ1

1 2729 gene gene SLC52A3

1 142 exon gene SLC52A3

143 919 exon gene SLC52A3

353 1762 CDS product SLC52A3a

note isoform a is encoded by transcript variant 1

920 1425 exon gene SLC52A3

1426 1549 exon gene SLC52A3

1550 2729 exon gene SLC52A3

ORIGIN

1 AAGTCCCAGC CCTGTGAGGG AATTCCGTGT GTGGGAACCG TGGGGAGGAG CTGCCAGGAT

61 TCAGGAGTTT CCTGGGTGAG GCGTGGCCAC AACGGACACT CCTGCTTTGT ACTAGAAGGA

121 AGAAGTATGG AGTTAAAGAC TGCAGCGTGA ACTGAGGAGT CCCGGACAGG CCGCTTGCTG

181 CAGAGGATCC AGTCCAGATC CCAGGAGAGC CCCTCTGCCC CTTCGGACCT CGTCTCCCAT

241 CTACAAAACG TGAAGATTGG CCCAGTTAGC GTGTCTCTAC AAAAAGGTGC ATATACCACT

301 GCCCCGCTGC AGGCTGATCT GAGAAAGCCT CTGGCCCAGG GCAGATACCG CCATGGCCTT

361 CCTGATGCAC CTGCTGGTCT GCGTCTTCGG AATGGGCTCC TGGGTGACCA TCAATGGGCT

421 CTGGGTAGAG CTGCCCCTGC TGGTGATGGA GCTGCCCGAG GGCTGGTACC TGCCCTCCTA

481 CCTCACGGTG GTCATCCAGC TGGCCAACAT CGGGCCCCTC CTGGTCACCC TGCTCCATCA

541 CTTCCGGCCC AGCTGCCTTT CCGAAGTGCC CATCATCTTC ACCCTGCTGG GCGTGGGAAC

601 CGTCACCTGC ATCATCTTTG CCTTCCTCTG GAATATGACC TCCTGGGTGC TGGACGGCCA

661 CCACAGCATC GCCTTCTTGG TCCTCACCTT CTTCCTGGCC CTGGTGGACT GCACCTCTTC

721 AGTGACCTTC CTGCCGTTCA TGAGCCGGCT GCCCACCTAC TACCTCACCA CCTTCTTTGT

781 GGGTGAAGGA CTCAGCGGCC TCTTGCCCGC CCTGGTGGCT CTTGCCCAGG GCTCCGGTCT

841 CACTACCTGC GTCAATGTCA CTGAGATATC AGACAGCGTA CCAAGCCCTG TACCCACGAG

901 GGAGACTGAC ATCGCACAGG GAGTTCCCAG AGCTTTGGTG TCCGCCCTCC CCGGAATGGA

961 AGCACCCTTG TCCCACCTGG AGAGCCGCTA CCTTCCCGCC CACTTCTCAC CCCTGGTCTT

1021 CTTCCTCCTC CTATCCATCA TGATGGCCTG CTGCCTCGTG GCGTTCTTTG TCCTCCAGCG

1081 TCAACCCAGG TGCTGGGAGG CTTCCGTGGA AGACCTCCTC AATGACCAGG TCACCCTCCA

1141 CTCCATCCGG CCGCGGGAAG AGAATGACTT GGGCCCTGCA GGCACGGTGG ACAGCAGCCA

1201 GGGCCAGGGG TATCTAGAGG AGAAAGCAGC CCCCTGCTGC CCGGCGCACC TGGCCTTCAT

1261 CTATACCCTG GTGGCCTTCG TCAACGCGCT CACCAACGGC ATGCTGCCCT CTGTGCAGAC

1321 CTACTCCTGC CTGTCCTATG GGCCAGTTGC CTACCACCTG GCTGCCACCC TCAGCATTGT

1381 GGCCAACCCT CTTGCCTCGT TGGTCTCCAT GTTCCTGCCT AACAGGTCTC TGCTGTTCCT

1441 GGGGGTCCTC TCCGTGCTTG GGACCTGCTT TGGGGGCTAC AACATGGCCA TGGCGGTGAT

1501 GAGCCCCTGC CCCCTCTTGC AGGGCCACTG GGGTGGGGAA GTCCTCATTG TGGCCTCGTG

1561 GGTGCTTTTC AGCGGCTGCC TCAGTTACGT CAAGGTGATG CTGGGCGTGG TCCTGCGCGA

1621 CCTCAGCCGC AGCGCCCTCT TGTGGTGCGG GGCGGCGGTG CAGCTGGGCT CGCTGCTCGG

1681 AGCGCTGCTC ATGTTCCCTC TGGTCAACGT GCTGCGGCTC TTCTCGTCCG CGGACTTCTG

1741 CAATCTGCAC TGTCCAGCCT AGGCAGGCCG CCGACCCCGC CCCCATCGCT CACGGACGGA

1801 ACTGGGGTCC AGAGAGGCCA GGTCACAGAG CAAGGGGCAG GAACAGAGAG ACAGAGCCTG

1861 AGTAATTGAA TCATGAACGC AAGTGCCCAC TGGGGACTGT GGGGAAGATG GCACCTGGAA

1921 ATGCAAGGTG CGGCTCTATC CCCAACTCTG TGTCACACTA CCTGTGACGA CCAGCTCAGA

1981 TCTCCTTTGC TTTGACTCTC AAGAGAGGAC TGATTTGCAG CATCTAGCTG GAGGCAGGCC

2041 CAAGGGTGTT AGAAGGGAAA CAGCTGGGAC AGCCGGCTGT CCCTTCAGGC TGTGTGACCT

2101 TGGGAAAGTC ATTTGGCTTC TCTGTGCCTG TTTCTTCATG CATGCAGTGG GGATTCCAGT

2161 AAGTACCAAC TACCTCACAG GCATGGCACG GAGGCAAAAG GAAAAAGCAG CCCGCATCAA

2221 GCAAGCCCTC CTGGGCCACC TGCTGATCTG ACAGTCCATC GTAGTAACAA GAGTGGCAGT

2281 CTGCACAACC TAGAAGTGGC CAGAAGGGTT GAGACACGCC CCTGCCCTCT CTCCTTTGCC

2341 CCTCAGTCTC ACAGAGGGGC TTCTACAAGA CAAGCAGATA ACGATAGAAT CTTGGGCATC

2401 TTGGCTTTCG GATTCTCAGT GTGGAGGGAC GTAGTACCCC ACACACCCCT TCCTGTCATC

2461 CTTCCTGGCC CATAAAGCCC ACTAGTTGGA GAGTAAGTAC CCTCCTGGAA GCCGGGAGAG

2521 ATGATTTGCT GGTGGGGCTG GGGAAGGCCC ATCCCTGAGC CTCTGAAAGT GAACTCCCCG

2581 ACCAGGTTGG GGACCAGACA TGCAGAGCCC CTGGAAGTAT TCTCTCAAAT GGAGGCAACA

2641 GAGGTGATTG TTATTTTGTT TTAGTTTCTG TTTTTCATTT TTTTAAATAA AGGCATTCCC

2701 TGCTTTTAAA AAAAAAAAAA AAAAAAAAA

**Supplementary Figure S2**

**Homo sapiens solute carrier family 52 member 3b (SLC52A3b), mRNA**

GenBank accession [No. KY978479](https://www.ncbi.nlm.nih.gov/nuccore/KY978479).

>SEQ2 [organism=Homo sapiens] Homo sapiens solute carrier family 52 member 3 (SLC52A3), transcript variant 2, mRNA, SLC52A3b

>Feature SEQ2

1 3073 gene gene SLC52A3

1 204 exon gene SLC52A3

205 822 exon gene SLC52A3

256 1503 CDS product SLC52A3b

note isoform a is encoded by transcript variant 2

823 1328 exon gene SLC52A3

1329 3073 exon gene SLC52A3

ORIGIN

1 ACTCCTGCTT TGTACTAGAA GGAAGAAGTA TGGAGTTAAA GACTGCAGCG TGAACTGAGG

61 AGTCCCGGAC AGGCCGCTTG CTGCAGAGGA TCCAGTCCAG ATCCCAGGAG AGCCCCTCTG

121 CCCCTTCGGA CCTCGTCTCC CATCTACAAA ACGTGAAGAT TGGCCCAGTT AGCGTGTCTC

181 TACAAAAAGG TGCATATACC ACTGCCCCGC TGCAGGCTGA TCTGAGAAAG CCTCTGGCCC

241 AGGGCAGATA CCGCCATGGC CTTCCTGATG CACCTGCTGG TCTGCGTCTT CGGAATGGGC

301 TCCTGGGTGA CCATCAATGG GCTCTGGGTA GAGCTGCCCC TGCTGGTGAT GGAGCTGCCC

361 GAGGGCTGGT ACCTGCCCTC CTACCTCACG GTGGTCATCC AGCTGGCCAA CATCGGGCCC

421 CTCCTGGTCA CCCTGCTCCA TCACTTCCGG CCCAGCTGCC TTTCCGAAGT GCCCATCATC

481 TTCACCCTGC TGGGCGTGGG AACCGTCACC TGCATCATCT TTGCCTTCCT CTGGAATATG

541 ACCTCCTGGG TGCTGGACGG CCACCACAGC ATCGCCTTCT TGGTCCTCAC CTTCTTCCTG

601 GCCCTGGTGG ACTGCACCTC TTCAGTGACC TTCCTGCCGT TCATGAGCCG GCTGCCCACC

661 TACTACCTCA CCACCTTCTT TGTGGGTGAA GGACTCAGCG GCCTCTTGCC CGCCCTGGTG

721 GCTCTTGCCC AGGGCTCCGG TCTCACTACC TGCGTCAATG TCACTGAGAT ATCAGACAGC

781 GTACCAAGCC CTGTACCCAC GAGGGAGACT GACATCGCAC AGGGAGTTCC CAGAGCTTTG

841 GTGTCCGCCC TCCCCGGAAT GGAAGCACCC TTGTCCCACC TGGAGAGCCG CTACCTTCCC

901 GCCCACTTCT CACCCCTGGT CTTCTTCCTC CTCCTATCCA TCATGATGGC CTGCTGCCTC

961 GTGGCGTTCT TTGTCCTCCA GCGTCAACCC AGGTGCTGGG AGGCTTCCGT GGAAGACCTC

1021 CTCAATGACC AGGTCACCCT CCACTCCATC CGGCCGCGGG AAGAGAATGA CTTGGGCCCT

1081 GCAGGCACGG TGGACAGCAG CCAGGGCCAG GGGTATCTAG AGGAGAAAGC AGCCCCCTGC

1141 TGCCCGGCGC ACCTGGCCTT CATCTATACC CTGGTGGCCT TCGTCAACGC GCTCACCAAC

1201 GGCATGCTGC CCTCTGTGCA GACCTACTCC TGCCTGTCCT ATGGGCCAGT TGCCTACCAC

1261 CTGGCTGCCA CCCTCAGCAT TGTGGCCAAC CCTCTTGCCT CGTTGGTCTC CATGTTCCTG

1321 CCTAACAGGT CTCTGCTGTT CCTGGGGGTC CTCTCCGTGC TTGGGACCTG CTTTGGGGGC

1381 TACAACATGG CCATGGCGGT GATGAGCCCC TGCCCCCTCT TGCAGGGCCA CTGGGGTGGG

1441 GAAGTCCTCA TTGTGAGTAT CCGGCCGGTG GGGCTGCTCC CGCTGCGTAC CCCTCACCCC

1501 TAGCCAAGGC CTGGGAGAGC TTGGGCGCGG CCCTTTCCAG CGAGCGGGGT CTGTGGGACC

1561 CTCCCGGGAA GCCCCAGGTG GGGGAAGCGC CCCTTCCTCA TTTGGGAGTG GAGGCTTAGG

1621 GAATCGAGGT GACTGGTAGG CCTCTTCCCA CCATGTGTAC CTCAGCAGGG GCCTTCTCTC

1681 AAGGGCCCTT GCCCTGATCA TGGGCACGTG AACCCAGAAA AGCCACTGGA TTCTCCTTCG

1741 CCCCCGGGCG GCCAACTCAT GGGACCCGTC GGCCCCGCAG GCTCACTCCC AGAAGGCGCT

1801 CCTCGCACCT AGAGAGTATT CCGTGGGTTC CCCTGAGCCC TGTGAGAGTT CTTTGCTCCG

1861 GCGCTCGCCC CGCAAGCCCC GTGACTCTGC ACCTCTTCCC TCCCGGCCCC GCAGGTGGCC

1921 TCGTGGGTGC TTTTCAGCGG CTGCCTCAGT TACGTCAAGG TGATGCTGGG CGTGGTCCTG

1981 CGCGACCTCA GCCGCAGCGC CCTCTTGTGG TGCGGGGCGG CGGTGCAGCT GGGCTCGCTG

2041 CTCGGAGCGC TGCTCATGTT CCCTCTGGTC AACGTGCTGC GGCTCTTCTC GTCCGCGGAC

2101 TTCTGCAATC TGCACTGTCC AGCCTAGGCA GGCCGCCGAC CCCGCCCCCA TCGCTCACGG

2161 ACGGAACTGG GGTCCAGAGA GGCCAGGTCA CAGAGCAAGG GGCAGGAACA GAGAGACAGA

2221 GCCTGAGTAA TTGAATCATG AACGCAAGTG CCCACTGGGG ACTGTGGGGA AGATGGCACC

2281 TGGAAATGCA AGGTGCGGCT CTATCCCCAA CTCTGTGTCA CACTACCTGT GACGACCAGC

2341 TCAGATCTCC TTTGCTTTGA CTCTCAAGAG AGGACTGATT TGCAGCATCT AGCTGGAGGC

2401 AGGCCCAAGG GTGTTAGAAG GGAAACAGCT GGGACAGCCG GCTGTCCCTT CAGGCTGTGT

2461 GACCTTGGGA AAGTCATTTG GCTTCTCTGT GCCTGTTTCT TCATGCATGC AGTGGGGATT

2521 CCAGTAAGTA CCAACTACCT CACAGGCATG GCACGGAGGC AAAAGGAAAA AGCAGCCCGC

2581 ATCAAGCAAG CCCTCCTGGG CCACCTGCTG ATCTGACAGT CCATCGTAGT AACAAGAGTG

2641 GCAGTCTGCA CAACCTAGAA GTGGCCAGAA GGGTTGAGAC ACGCCCCTGC CCTCTCTCCT

2701 TTGCCCCTCA GTCTCACAGA GGGGCTTCTA CAAGACAAGC AGATAACGAT AGAATCTTGG

2761 GCATCTTGGC TTTCGGATTC TCAGTGTGGA GGGACGTAGT ACCCCACACA CCCCTTCCTG

2821 TCATCCTTCC TGGCCCATAA AGCCCACTAG TTGGAGAGTA AGTACCCTCC TGGAAGCCGG

2881 GAGAGATGAT TTGCTGGTGG GGCTGGGGAA GGCCCATCCC TGAGCCTCTG AAAGTGAACT

2941 CCCCGACCAG GTTGGGGACC AGACATGCAG AGCCCCTGGA AGTATTCTCT CAAATGGAGG

3001 CAACAGAGGT GATTGTTATT TTGTTTTAGT TTCTGTTTTT CATTTTTTTA AATAAAGGCA

3061 TTCCCTGCTT TTA

**Supplementary Figure S3**


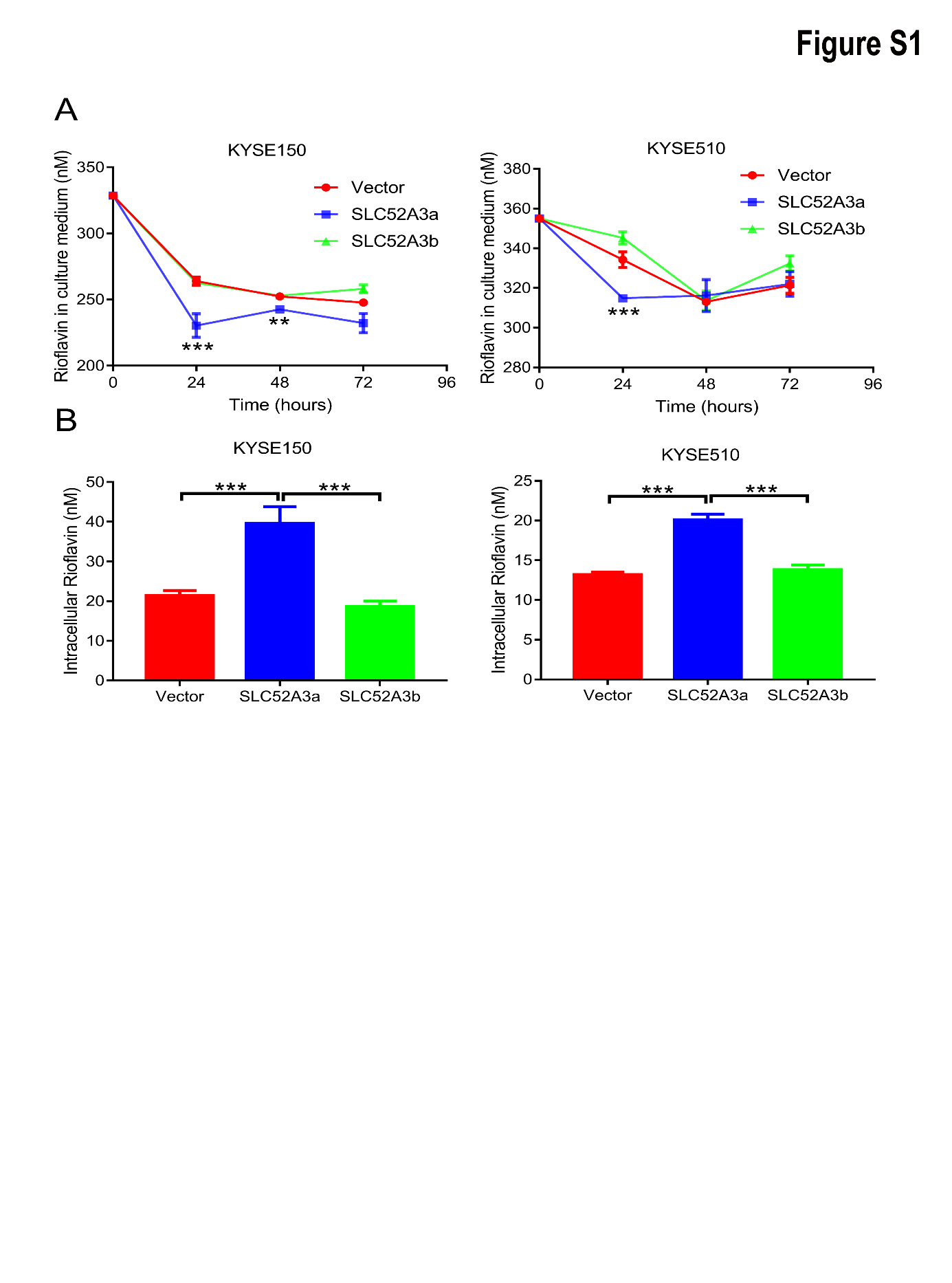


**Supplementary Fig. S3** Concentrations of riboflavin in culture medium and intracellular were quantified by HPLC. **a** The concentrations of riboflavin in culture medium after KYSE150 and KYSE510 cells were being transfected with vector, SLC52A3a or SLC52A3b for 0 h, 24 h, 48 h and 72 h. **b** The intracellular riboflavin concentrations in KYSE150 and KYSE510 after being transfected with vector, SLC52A3a or SLC52A3b for 72 h. The value was an average of three times repeats, and error bars indicate S.D. ***P*<0.01, ****P*<0.001 based on Student’s t-tests.

| **Supplementary Table S1** The clinical characteristics and survival information of the patients for the immunohistochemical staining (Shantou Central Hospital from 1987 to 1997, 246 cases). | | | | |
| --- | --- | --- | --- | --- |
| Characteristics | | Case No. | 5-year survival rate (%) | *P** |
| Total no. (deceased: 131) | | 246 | 40.8 |  |
| Mean age (range) | | 54.5 (32-73) yr |  |  |
| Median follow-up (range) | | 25.2 (1.1-133.0) months |  |  |
| Gender | female | 71 | 40 | 0.975 |
|  | male | 175 | 41 |  |
| Therapies received | Only surgery | 165 | 43.2 | 0.494 |
|  | Surgery + chemotherapy | 47 | 30.5 |  |
|  | Surgery + radiotherapy | 29 | 45.8 |  |
|  | Surgery+ chemo+ radio | 5 | 0 |  |
| Histological grade | well-differentiation | 56 | 56.4 | 0.044 |
|  | moderate-differentiation | 154 | 38.3 |  |
|  | poor-differentiation | 36 | 27.6 |  |
| Tumor size | <5 cm | 140 | 43.6 | 0.047 |
|  | ≥5 cm | 105 | 36.1 |  |
| Depth of tumor invasion | T1 or T2 | 1 + 42 | 51.9 | 0.062 |
|  | T3 or T4 | 199 + 4 | 38.5 |  |
| Regional lymph nodes | N0 | 153 | 51.2 | 0.000 |
|  | N1 | 93 | 23.3 |  |
| Distant metastasis | M0 | 238 | 41.9 | 0.026 |
|  | M1 | 8 | 12.5 |  |
| pTNM stages | I or II | 1 + 156 | 50.8 | 0.000 |
|  | III or IV | 81+ 8 | 22.9 |  |
| Chem, chemotherapy; radio, radiotherapy. | | | | |
| *Log-rank test of Kaplan Meier method; *P* <0.05 was considered significant. | | | | |

| **Supplementary Table S2** The clinicopathological characteristics of generation dataset of patients with ESCC (Shantou Central Hospital from 2007 to 2014, 290 cases). | | | | | |
| --- | --- | --- | --- | --- | --- |
| Characteristics | | Case No. | 3-year survival rate (%) | 5-year survival rate (%) | *P** |
| Total no. | | 290 |  |  |  |
| Mean age (year) | | 58.7 |  |  |  |
| Gender | female | 61 | 47.5 | 37.2 | 0.381 |
|  | male | 229 | 50.1 | 41.4 |  |
| Therapies received | Only surgery | 158 | 48.0 | 41.2 | 0.281 |
|  | Surgery + chemotherapy | 59 | 50.8 | 42.0 |  |
|  | Surgery + radiotherapy | 43 | 58.1 | 46.3 |  |
|  | Surgery+ chemo+ radio | 28 | 34.6 | 19.2 |  |
| Histological grade | G1 | 42 | 64.3 | 54.7 | 0.000 |
|  | G2 | 222 | 49.6 | 40.6 |  |
|  | G3 | 26 | 24.0 | 16.0 |  |
| Tumor size | <3 cm | 66 | 50.3 | 46.8 | 0.099 |
|  | 3-5 cm | 137 | 48.1 | 40.6 |  |
|  | ≥5 cm | 87 | 43.7 | 33.0 |  |
| Depth of tumor invasion | T1 | 10 | 66.7 | 66.7 | 0.349 |
|  | T2 | 48 | 49.0 | 40.5 |  |
|  | T3 | 231 | 49.3 | 39.8 |  |
|  | T4 | 1 | - | - |  |
| Regional lymph nodes | N0 | 148 | 61.6 | 53.0 | 0.000 |
|  | N1 | 76 | 51.2 | 45.6 |  |
|  | N2 | 48 | 31.2 | 12.5 |  |
|  | N3 | 18 | 5.6 | - |  |
| pTNM stages | Ⅰ | 20 | 79.2 | 73.9 | 0.000 |
|  | Ⅱ | 143 | 57.7 | 48.8 |  |
|  | Ⅲ | 127 | 36.0 | 26.2 |  |
| Chem, chemotherapy; radio, radiotherapy. | | | | | |
| *Log-rank test of Kaplan Meier method; *P* <0.05 was considered significant. | | | | | |

| **Supplementary Table S3** PCR primers used in this study | | |
| --- | --- | --- |
| **Primer** | **Sequence（5**′**-3**′**）** | **Position** |
|  | **Primers for 5'RACE** |  |
| SLC52A3-5′RACE-GSP1 | CAGTGACATTGACGCAGGTAGTGA | 730 ~ 753 (exon 2) |
| SLC52A3-5′RACE-GSP2 | GAGGACCAAGAAGGCGATGC | 556 ~ 575(exon 2) |
| SLC52A3-5′RACE-GSP3 | GCGTGTCTCAACCCTTCTGG | 2190 ~ 2209(exon 5) |
| SLC52A3-5′RACE-GSP4 | CAGACTGCCACTCTTGTTACTACGAT | 2148 ~ 2173(exon 5) |
|  | **Primers for cloning** |  |
| SLC52A3-F | GGATCCATTGGCCCAGTTAGCGTGTC | -97 ~ -78 (exon 1) |
| SLC52A3-R | GAATTCGCCGCACCTTGCATTTCC | 1565 ~ 1582 (exon 5) |
|  | **Primers for luciferase-reporter gene constructs** |  |
| SLC52A3-5′FR-1F | CCGCTCGAG CTTTCCCTGTTTGCCAGAAGGAATACCC | -5076 ~ -5049 |
| SLC52A3-5′FR-2F | CCGCTCGAGTTGAATGCAACTGATACGTTTGCTGAAAT | -3825 ~ -3797 |
| SLC52A3-5′FR-3F | CCGCTCGAGTGGCAGTGGCCAGTGAGTTAACAATTTA | -3391 ~ -3364 |
| SLC52A3-5′FR-4F | CCGCTCGAGTTCGCTCAGTGAAGGTATGAGGAAGGAA | -2849 ~ -2822 |
| SLC52A3-5′FR-5F | CCGCTCGAGAAAAAGCTTCAGTCTCAAGGTATGTGC | -3288 ~ -3263 |
| SLC52A3-5′FR-6F | CCGCTCGAGTCACCTGTGCTATTAGTCAAGTCAG | -3020 ~ -2995 |
| SLC52A3-5′FR-7F | CCGCTCGAGCTAGGGGATCTGACTGCGTTACAC | -2935 ~ -2912 |
| SLC52A3-5′FR-8F | CCGCTCGAGCTCCCAGAGAGGAAAAGGAGGTG | -2897 ~ -2875 |
| SLC52A3-5′FR-1R | GGAAGATCTCGTGAAATGGCAGGTTTGGTGACATCGT | -2430 ~ -2403 |
| SLC52A3-5′FR-2R | GGAAGATCTCCCCTACTAGGTATCAAGTGAACTC | -2519 ~ -2495 |
| SLC52A3-5′FR-3R | GGAAGATCTATGGGAGACGAGGTCCGAAG | -2602 ~ -2583 |
| SLC52A3-5′FR-4R | GGAAGATCTTTCACGCTGCAGTCTTTAACTC | -2693 ~ -2672 |
| SLC52A3-5′FR-5R | GGAAGATCTCTCACCCAGGAAACTCCTGAA | -2763 ~ -2743 |
| SLC52A3-5′FR-6R | GGAAGATCTACGGTTCCCACACACGGAATTCC | -2804 ~ -2782 |
|  | **Primers for ChIP** |  |
| ChIP-F | CTAGGGGATCTGACTGCGTTACAC | -2935 ~ -2912 |
| ChIP-R | ATGGGAGACGAGGTCCGAAG | -2602 ~ -2583 |
|  | **Probes for EMSA** |  |
| Top | TGGGGAGGAGCTGCCAGGATTCAGGAGTTTCCTGGGTGAG | -2782~-2743 |
| Bottom | CTCACCCAGGAAACTCCTGAATCCTGGCAGCTCCTCCCCA | -2782~-2743 |
|  | **Primers for qRT-PCR** |  |
| SLC52A3a-qF | CCTCGTTGGTCTCCATGTTCCTG | exon 3 |
| SLC52A3a-qR | GCCGCTGAAAAGCACCCACG | exon 5 |
| SLC52A3b-qF | CCTCGTTGGTCTCCATGTTCCTG | exon 3 |
| SLC52A3b-qR | CCCCACCGGCCGGATACTCA | exon 4 |
| ACTB-qF | CAACTGGGACGACATGGAGAAA | exon 3 |
| ACTB-qR | GATAGCAACGTACATGGCTGGG | exon 4 |
| F: forward primer; R: reverse primer. | | |
| Cutting sites are underlined. | | |
